# Targeted sequencing identifies a novel *SH2D1A* pathogenic variant in a Chinese family: Carrier screening and prenatal genetic testing

**DOI:** 10.1371/journal.pone.0172173

**Published:** 2017-02-23

**Authors:** Jun-Yu Zhang, Song-Chang Chen, Yi-Yao Chen, Shu-Yuan Li, Lan-Lan Zhang, Ying-Hua Shen, Chun-Xin Chang, Yu-Qian Xiang, He-Feng Huang, Chen-Ming Xu

**Affiliations:** 1 The International Peace Maternity & Child Health Hospital Affiliated to Shanghai Jiao Tong University School of Medicine, Shanghai, P. R. China; 2 Institute of Embryo-Fetal Original Adult Disease Affiliated to Shanghai Jiao Tong University School of Medicine, Shanghai, P. R. China; IMAGINE, FRANCE

## Abstract

X-linked lymphoproliferative disease type 1 (XLP1) is a rare primary immunodeficiency characterized by a clinical triad consisting of severe EBV-induced hemophagocytic lymphohistiocytosis, B-cell lymphoma, and dysgammaglobulinemia. Mutations in *SH2D1A* gene have been revealed as the cause of XLP1. In this study, a pregnant woman with recurrence history of birthing immunodeficiency was screened for pathogenic variant because the proband sample was unavailable. We aimed to clarify the genetic diagnosis and provide prenatal testing for the family. Next-generation sequencing (NGS)-based multigene panel was used in carrier screening of the pregnant woman. Variants of immunodeficiency related genes were analyzed and prioritized. Candidate variant was verified by using Sanger sequencing. The possible influence of the identified variant was evaluated through RNA assay. Amniocentesis, karyotyping, and Sanger sequencing were performed for prenatal testing. We identified a novel *de novo* frameshift *SH2D1A* pathogenic variant (c.251_255delTTTCA) in the pregnant carrier. Peripheral blood RNA assay indicated that the mutant transcript could escape nonsense-mediated mRNA decay (NMD) and might encode a C-terminal truncated protein. Information of the variant led to success prenatal diagnosis of the fetus. In conclusion, our study clarified the genetic diagnosis and altered disease prevention for a pregnant carrier of XLP1.

## Introduction

X-linked lymphoproliferative disease type 1 (XLP1; OMIM 308240), also known as Duncan’s disease, is a rare primary immunodeficiency characterized by exquisite sensitivity to Epstein-Barr virus (EBV) infection. It has an incidence of approximately 1–3 per million in male births [1]. Clinical manifestations of XLP1 are varied and usually include a clinical triad consisting of severe EBV-induced hemophagocytic lymphohistiocytosis (HLH), B-cell lymphoma, and dysgammaglobulinemia [2].

Loss-of-function pathogenic variants in *SH2D1A* gene had been linked to XLP1 [3–5]. *SH2D1A* gene, located on chromosome Xq25, consists of 4 exons and encodes a 128-amino acid intracellular SLAM (signaling lymphocytic activation molecule)-associated protein (SAP). SAP consists of an N-terminal region (5 aa), a C-terminal region (~20 aa) and ~100 aa central Src homology 2 (SH2) domain, and is expressed in T cells, natural killer (NK) cells, NKT cells, platelets, eosinophils, and some B-cell populations [6]. Through the interaction with SLAM family of immunomodulatory receptors (SLAM, 2B4, CD84, Ly9, CD84, NTBA and CRACC), SAP plays key roles in regulating lymphocyte adhesion and interactions, which are required for the normal development, homeostasis and immune system function [6, 7].

The *SH2D1A* is extremely conserved among species and found to be highly nonpolymorphic [4, 8]. To date, over 100 *SH2D1A* mutations have been included in HGMD (the Human Gene Mutation Database) [9]. Although it has been appreciated for nearly two decades that mutation of *SH1D1A* result in XLP1, the management of XLP1 is still difficult and death usually occurs within 2 months from patients presenting with EBV-induced HLH [10]. Hematopoietic stem cell transplantation has been considered as the only treatment against XLP1 [11], just like many primary immunodeficiency diseases. The mean age at death reported for individuals with *SH2D1A* pathogenic variant is 11 years (ranging from 2 years to 69 years) [12].

The use of next-generation sequencing (NGS) technology to move from testing small panels of genes to large multi-gene panels made its clinical application possible. Performing a larger panel but then restricting analysis to a disease-associated set of genes based on the subject’s clinical phenotypes has been recommend to be more efficient in clinical diagnosis [13].

In this study, we reported the identification of a novel frameshift *SH2D1A* pathogenic variant in a pregnant carrier of a Chinese family. The consequence of the variant was analyzed through RNA assay. Moreover, the potential transmitting of the variant was prenatal tested by use of fetal DNA derived from amniocytes.

## Materials and methods

### Subjects and ethics statement

**Fig 1:**
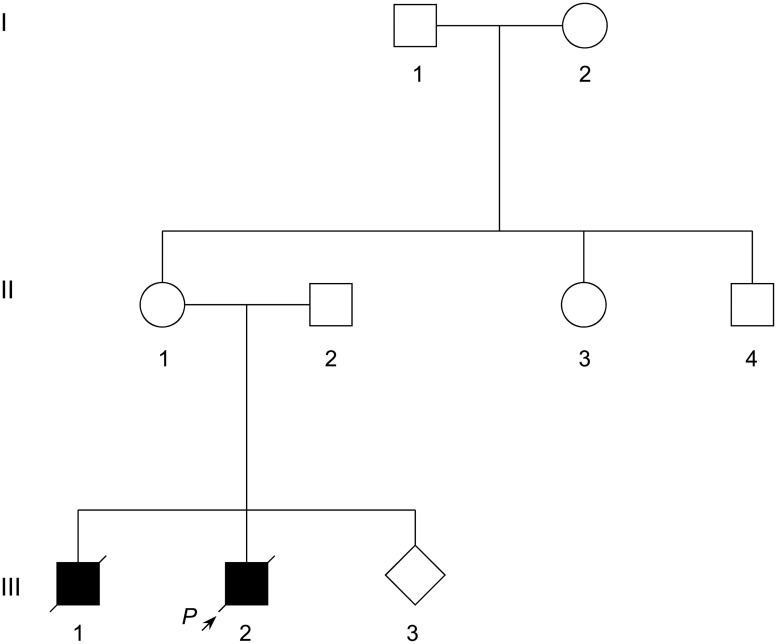
Pedigree of a Chinese family with risk for recurrence of immunodeficiency. Arrow indicates the proband. Alive family members were subjected to genetic analysis.

All the subjects of this study were recruited from the outpatient department of the International Peace Maternity & Child Health Hospital (IPMCH), Shanghai Jiao Tong University School of Medicine. Peripheral blood samples were collected from all members of the pedigree (Fig 1) as well as 192 ethnically matched unrelated healthy female controls. Genomic DNA was extracted according to standard techniques. This study was prospectively reviewed and approved by the Ethics Review Committee of IPMCH of Shanghai Jiao Tong University School of Medicine, and conducted according to the Declaration of Helsinki Principles. Written informed consents were obtained from all participants, including 192 healthy female control individuals.

### Targeted sequence capture and next-generation sequencing

Sequencing library preparation and target capture had been described in detail previously [14]. Briefly, DNA was sheared to create fragments of 200 to 250 bp. We captured target regions of 2181 genes associated with 561 Mendelian diseases. The total size of target regions of the capture array was 6.19 Mb. Paired-end sequencing (2×90 bp) was performed with Illumina HiSeq2500 Analyzers (Illumina, San Diego, CA, USA) to generate average sequence coverage of more than 100× with more than 97% of target bases having at least 10× coverage.

### Data filtering, mapping and variant detection

Sequencing output data were converted from bcl files into FastQ files by using the Illumina consensus assessment of sequence and variation (CASAVA) software. Alignment of sequence reads to the human reference genome (hg19) was performed by using Burrows-Wheeler Aligner (BWA; v.0.7.12)[15]. The Genome Analysis Toolkit (GATK; v.3.5) was applied for variants calling. SNVs and indels were annotated with ANNOVAR[16] and prioritized with in-house developed scripts (MultiOmics One) restricted to immunodeficiency related genes (*ADA*, *AK2*, *ATM*, *BLNK*, *BTK*, *CD19*, *CD3D*, *CD3E*, *CD3G*, *CD79A*, *CD79B*, *CD81*, *CORO1A*, *CR2*, *DCLRE1C*, *ICOS*, *IKBKG*, *IL2RG*, *IL7R*, *JAK3*, *LIG4*, *LRBA*, *MS4A1*, *NFKBIA*, *PNP*, *PTPRC*, *RAG1*, *RAG2*, *SH2D1A*, *SP110*, *TNFRSF13B*, *TNFRSF13C*, *XIAP*, and *ZAP70*).

### Variant confirmation by Sanger sequencing

Prioritized candidate variants were confirmed by Sanger sequencing. The primers for verifying *SH2D1A* variant (SH2D1A-F: 5’-TCTCTTAGCATCCCTAGCACA-3’ and SH2D1A-R: 5’-TGTGTACTTCTAGCTGAGGACT-3’) were designed by using Primer3web[17]. All family members and 192 female control individuals were analyzed. Variant nomenclature was based on published suggestions[18].

### RT-PCR analysis of *SH2D1A* mRNA in peripheral blood mononuclear cells

Total RNA from peripheral blood mononuclear cells (PBMCs) were extracted and reversely transcribed from the carrier (II-1) and a healthy control. The RT-PCR were performed with the primers SH2D1A-RT-F: 5’-CCAGGCGTGTACTGCCTATG-3’ and SH2D1A-RT-R: AGCTGAGGACTTCTTCTCAACTG. The amplified DNA fragments were directly subcloned into the TA cloning vector pGM-T (TIANGEN Biotech, Beijing, China) and sequenced by using the universal sequencing T7 primer.

### Identity testing

A short tandem repeat (STR) typing human personal identification detection kit (R1004T; GENESKY, Shanghai, China) was used for identity testing in *de novo* variant confirmation and potential maternity contamination analysis in prenatal testing, according to the instruction manuals. The amplicons were analyzed by using Applied Biosystems 3500Dx sequencer.

### Prenatal testing

Amniocentesis was performed in 18 weeks of gestation using a 22-gauge spinal needle and continuous ultrasonographic guidance [19]. An amniotic fluid sample of 30 mL was obtained. Chromosomal karyotyping and sanger sequencing were performed in parallel.

## Results

### Clinical characteristics of a Chinese immunodeficiency family

A healthy 38-year-old pregnant woman of 8 weeks’ gestation, with twice abnormal reproduction history, was referred to reproductive genetics clinic for counseling regarding her risk for having a child with immunodeficiency. The pedigree comprised 9 individuals spanning three generations (Fig 1). The proband boy (III-2) and his elder brother (III-1) were both diagnosed with immunodeficiency, and died with rapid disease progression from EBV infection, bronchopneumonia, severe anemia, and multiple organ dysfunction at around 2 years of age. Unfortunately, no DNA analysis had been conducted on the proband and his brother to determine which type of pathogenic gene they had. No other family member had this disorder. Family histories of immunodeficiency and abnormal reproduction were denied. The clinical features of the family supported a provisional diagnosis of immunodeficiency disease.

### Identification of a novel *SH2D1A* pathogenic variant by NGS-based carrier screening

**Fig 2:**
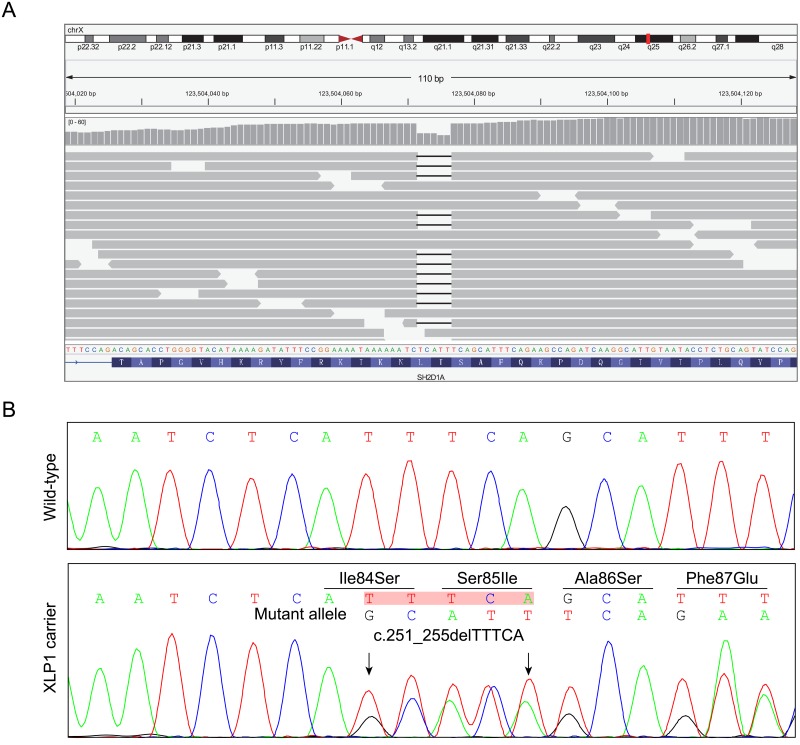
Identification of a novel *SH2D1A* pathogenic variant in the pregnant woman. (A) Integrative Genomics Viewer (IGV) snapshot of the sequence data at *SH2D1A* for the pregnant (II-1) sample, and the corresponding calls made from the data. Each horizontal gray bar represents one read, the short line in the gray bar indicates bases deletion. (B) Sanger sequencing verified the NGS result. The arrows indicate the deletion of TTTCA in mutant allele which leads to frameshift and introduces a premature termination codon (p.Ile84Serfs*18).

No proband sample was preserved, direct proband genetic diagnosis was prevented, consequently. On the basis of the clinical features of the family, NGS-based carrier screening of 2181 genes including 34 immunodeficiency-related genes was carried out with peripheral blood of the pregnant woman (II-2). Totally, 2.4 Gb raw data was obtained, with a mean coverage of 122.99× and 97.7% of bases in the defined target reached at least 10× coverage. Date priority filtering revealed that the pregnant woman was heterozygous for a novel deletion variant NM_002351.4(*SH2D1A*):c.[251_255delTTTCA];[251_255TTTCA=] (Fig 2A).

Subsequent Sanger sequencing of the variant-containing PCR amplicons was performed. Consistent with the NGS results, the variant was detected in the pregnant woman (Fig 2B). Notably, the variant was not detected in other alive family members, including the parents of the pregnant women. Identity testing of both parental samples using short tandem repeat (STR) loci and amelogenin confirmed the *de novo* characteristics (Table 1), which was a strong evidence of pathogenicity according to ACMG guideline [20].

**Table 1:** Short tandem repeat typing results for the members of the pedigree.

Marker	I-1		I-2		II-1		II-2		III-3	
	Allele 1	Allele 2	Allele 1	Allele 2	Allele 1	Allele 2	Allele 1	Allele 2	Allele 1	Allele 2
Amelo	X	Y	X	X	X	X	X	Y	X	Y
D13S317	9	12	12	12	12	12	8	12	12	12
D7S820	8	12	10	11	8	10	10	12	8	12
G4S0001	13	17	14	16	14	17	14	14	14	17
G2S0002	16	18	16	23	16	16	16	24	16	16
D18S51	14	17	14	22	14	14	16	17	14	16
D8S1179	10	13	14	15	10	14	12	15	14	15
D2S1338	18	20	23	24	20	23	17	19	17	20
G15S0001	12	12	12	12	12	12	10	14	10	12
D16S539	10	12	11	15	11	12	9	11	9	11
VWA	14	14	14	17	14	14	14	18	14	18
G7S0005	7	9	9	10	7	9	10	10	9	10
G10S0001	18	23	19	19	18	19	19	19	19	19
THO1	6	9	7	9	7	9	9	9	7	9
D8S588	8	8	11	12	8	12	13	13	12	13
G5S0001	7	9	9	10	7	9	10	11	7	10
D5S818	12	13	10	12	12	13	9	11	9	13

Note: The analysis was performed using a human identification kit that amplified 16 short tandem repeat loci and the amelogenin locus (for sex typing). Values shown are the numbers of short tandem repeats detected at each allele of each locus.

The variant was neither found in 192 unrelated female control subjects, nor included in public population databases, including Exome Aggregation Consortium (ExAC; http://exac.broadinstitute.org) [21] and 1000 Genomes Project (http://browser.1000genomes.org) [22]. It was predicted to cause a frameshift and produce a 17-amino acid extended polypeptide (p.Ile84Serfs*18), disrupting the functional SH2 domain of SAP. The predicted premature termination codon (PTC) is located in the penultimate exon of *SH2D1A*, 38-bp upstream of the last exon 3-exon 4 junction. Therefore, the corresponding mutant transcript could escape the nonsense-mediated mRNA decay (NMD) [23, 24].

### RT-PCR analysis of *SH2D1A* mRNA

**Fig 3:**
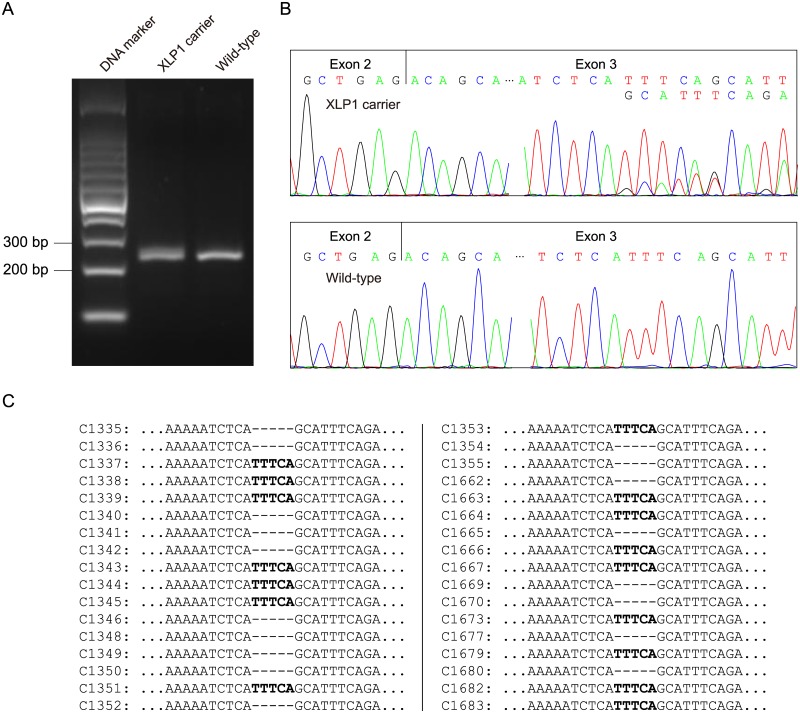
RNA assay of the SH2D1A mRNA. (A) RT-PCR analysis of SH2D1A mRNA from peripheral blood mononuclear cells (PBMCs) of the pregnant carrier (II-1) and a healthy female control. The cDNA fragments of 213-bp, which corresponded to the predicted wild-type transcript, were identified in both the pregnant carrier and the control. (B) cDNA sequencing revealed that *SH2D1A* transcripts of the pregnant carrier were heterozygous for c.251_255delTTTCA. (C) Sequencing analysis results of the pGM-T clones with the cDNA amplicons.

To evaluate the influence of c.251_255delTTTCA on *SH2D1A* mRNA, RT-PCR analysis of total RNA extracted from peripheral blood mononuclear cells (PBMCs) of the pregnant woman (II-1) and a healthy female control was performed with the primers of SH2D1A-RT-F and SH2D1A-RT-R. The same splicing pattern was found between the wild-type control and the c.251_255delTTTCA heterozygous pregnant woman (Fig 3A). Further cDNA sequencing revealed that *SH2D1A* transcripts of the pregnant carrier were heterozygous for c.251_255delTTTCA (Fig 3B). RT-PCR products were then subcloned into pGM-T vector. Totally, 34 clones were sequenced using T7 universal primer. Sequence analysis showed an approximately 1:1 proportion of wild-type and c.251_255delTTTCA clones (16:18) (Fig 3C). These results suggested that the c.251_255delTTTCA mutant transcripts could escape the NMD and might encode a C-terminal truncated protein.

### Variant classifying and prenatal testing

The variant can be classified into pathogenic variant according to ACMG guideline for sequence variants interpretation [20]. The family was therefore counseled that the risk for affected sons was 50%, the risk for carrier daughters was 50%, and the risk for affected daughters was low but dependent on the degree of skewing of X inactivation.

**Fig 4:**
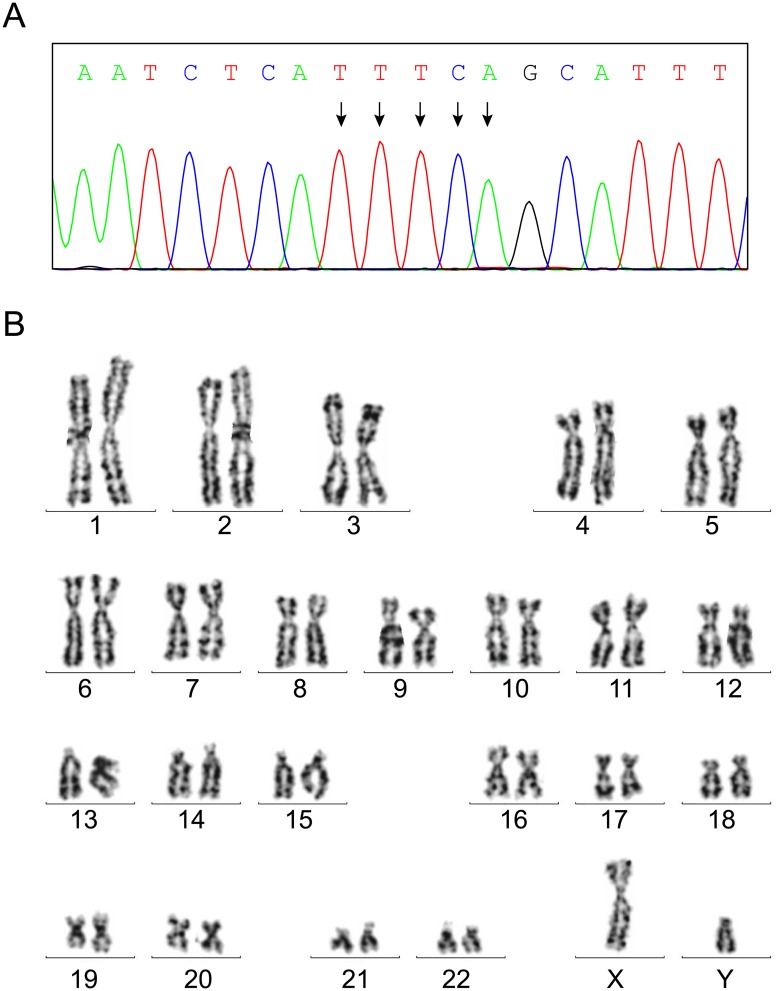
Prenatal testing of the fetus. (A) Sanger sequencing revealed that the fetus was unaffected. (B) Karyotyping result of the fetus was consistent with a normal 46,XY chromosomal constitution.

After determining the pathogenicity of the variant, amniocentesis was performed in 18 weeks of gestation of the pregnant carrier. Both variant analysis and chromosomal karyotyping were performed with amniotic fluid fetal cells (Fig 1, III-3). STR analysis ruled out the possibility that maternal blood would contaminate the sample on testing (Table 1). Sanger sequencing and karyotyping results demonstrated that the fetus (III-3) was unaffected (NM_002351.4(*SH2D1A*):c.[251_255delTTTCA];[0]) with normal male karyotype (Fig 4). Pregnancy with unaffected fetus continued after genetic counseling. The neonate was born at 40 weeks’ gestation after natural delivery with the Apgar score of 10 points.

## Discussion

The success in carrier screening and prenatal diagnosis of Tay-Sachs disease (TSD; OMIM 272800) in Ashkenazi Jewish population has promoted the application of the screening panel strategy in disease prevention [25]. Prenatal genetic testing can be performed by targeted genetics testing of fetal cells, thus providing the opportunity for pregnancy termination if the pathogenic variant in the affected family has been identified.

XLP is a rare but life-threaten disorder with unknown ethnic or racial predilection. Only a few cases of XLP were reported in Chinese population [26, 27]. Both *SH2D1A* and *XIAP* pathogenic variants were associated with XLP. Approximately, 83–97% XLP patients were attributed to pathogenic variants of *SH2D1A* gene and subclassified as XLP1; while 12% attributed to *XIAP* gene and subclassified as XLP2 (OMIM 300635)[28–30]. XLP1 is inherited in an X-linked recessive manner. *SH2D1A* pathogenic variant carrier females have a half chance of transmitting the pathogenic allele in each pregnancy. In this report, the lack of affected male proband sample brought about the necessity of female carrier screening for further disease prevention. The prenatal genetic diagnostic testing usually cannot be performed until the pathogenic variant has been identified, although NGS-based prenatal screening has been reported [31, 32]. Affected male proband is the most suitable for initial gene testing. For lethal or severe recessive diseases, however, proband usually dies in prenatal or neonatal periods. Thus, phenotype-based carrier screening prior to conception, likely in the form of exome or multigene panel sequencing, could give the family an opportunity for genetic diagnosis and birth defects prevention.

Apart from the risk for recurrence of XLP1 mentioned above, lymphoma was also been reported in *SH2D1A* female carrier due to nonrandom X-inactivation [33], suggesting that carrier females in an XLP kindred might be at the risk of developing XLP phenotype and should be monitored for early symptoms. Thus, screening *SH2D1A* may provide early molecular diagnosis for both XLP1 patients and the potential XLP1 carriers.

The *de novo* pathogenic variant of *SH2D1A* identified in this report has not been previously described. RNA assay indicated that the variant could escape NMD and might introduce a truncated protein. Mutations in *SH2D1A* are primarily loss-of-function result in diminished or absent protein expression [34]. There is no apparent genotype-phenotype correlation between *SH2D1A* mutations and severity of the disease. The most frequently reported mutation involved the Arginine at codon 55 (exon 2) in previous publications worldwide, as well as in China [10, 26].

In summary, we reported a family that the proband died neonatally with immunodeficiency features. We identified a novel *de novo* frameshift *SH2D1A* pathogenic variant (c.251_255delTTTCA) in the pregnant carrier by using multigene panel carrier screening. Peripheral blood RNA assay indicated that the mutant transcript could escape NMD and might encode an C-terminal truncated protein. Further, information of the variant led to successful prenatal diagnosis of the fetus. Our study clarified the clinical diagnosis and altered disease prevention for the family. The novel pathogenic variant identified in this report can enrich the mutation database of *SH2D1A*. Moreover, our approach of multigene panel carrier screening and prenatal genetic testing would provide valuable insights in genetic disease families with inheritance risk.

## References

[1] Purtilo DT, Grierson HL, Davis JR, Okano M. The X-linked lymphoproliferative disease: from autopsy toward cloning the gene 1975–1990. Pediatr Pathol. 1991;11(5):685–710. 1660601 10.3109/15513819109065466

[2] Tangye SG. XLP: clinical features and molecular etiology due to mutations in SH2D1A encoding SAP. J Clin Immunol. 2014;34(7):772–9. 10.1007/s10875-014-0083-7 25085526

[3] Coffey AJ, Brooksbank RA, Brandau O, Oohashi T, Howell GR, Bye JM, et al Host response to EBV infection in X-linked lymphoproliferative disease results from mutations in an SH2-domain encoding gene. Nat Genet. 1998;20(2):129–35. 10.1038/2424 9771704

[4] Sayos J, Wu C, Morra M, Wang N, Zhang X, Allen D, et al The X-linked lymphoproliferative-disease gene product SAP regulates signals induced through the co-receptor SLAM. Nature. 1998;395(6701):462–9. 10.1038/26683 9774102

[5] Nichols KE, Harkin DP, Levitz S, Krainer M, Kolquist KA, Genovese C, et al Inactivating mutations in an SH2 domain-encoding gene in X-linked lymphoproliferative syndrome. Proc Natl Acad Sci U S A. 1998;95(23):13765–70. 9811875 10.1073/pnas.95.23.13765 PMC24894

[6] Schwartzberg PL, Mueller KL, Qi H, Cannons JL. SLAM receptors and SAP influence lymphocyte interactions, development and function. Nature Reviews Immunology. 2009;9(1):39–46. 10.1038/nri2456 19079134

[7] Ma CS, Deenick EK. The role of SAP and SLAM family molecules in the humoral immune response. Ann N Y Acad Sci. 2011;1217:32–44. 10.1111/j.1749-6632.2010.05824.x 21091715

[8] Morra M, Howie D, Grande MS, Sayos J, Wang N, Wu C, et al X-linked lymphoproliferative disease: a progressive immunodeficiency. Annu Rev Immunol. 2001;19:657–82. 10.1146/annurev.immunol.19.1.657 11244050

[9] Stenson PD, Ball EV, Mort M, Phillips AD, Shiel JA, Thomas NS, et al Human Gene Mutation Database (HGMD): 2003 update. Hum Mutat. 2003;21(6):577–81. 10.1002/humu.10212 12754702

[10] Booth C, Gilmour KC, Veys P, Gennery AR, Slatter MA, Chapel H, et al X-linked lymphoproliferative disease due to SAP/SH2D1A deficiency: a multicenter study on the manifestations, management and outcome of the disease. Blood. 2011;117(1):53–62. 10.1182/blood-2010-06-284935 20926771 PMC3374620

[11] Lankester AC, Visser LF, Hartwig NG, Bredius RG, Gaspar HB, van der Burg M, et al Allogeneic stem cell transplantation in X-linked lymphoproliferative disease: two cases in one family and review of the literature. Bone Marrow Transplant. 2005;36(2):99–105. 10.1038/sj.bmt.1705016 15908972

[12] Pachlopnik Schmid J, Canioni D, Moshous D, Touzot F, Mahlaoui N, Hauck F, et al Clinical similarities and differences of patients with X-linked lymphoproliferative syndrome type 1 (XLP-1/SAP deficiency) versus type 2 (XLP-2/XIAP deficiency). Blood. 2011;117(5):1522–9. 10.1182/blood-2010-07-298372 21119115

[13] Rehm HL, Bale SJ, Bayrak-Toydemir P, Berg JS, Brown KK, Deignan JL, et al ACMG clinical laboratory standards for next-generation sequencing. Genet Med. 2013;15(9):733–47. 10.1038/gim.2013.92 23887774 PMC4098820

[14] Liu Y, Wei X, Kong X, Guo X, Sun Y, Man J, et al Targeted Next-Generation Sequencing for Clinical Diagnosis of 561 Mendelian Diseases. PLoS One. 2015;10(8):e0133636 10.1371/journal.pone.0133636 26274329 PMC4537117

[15] Li H, Durbin R. Fast and accurate short read alignment with Burrows-Wheeler transform. Bioinformatics. 2009;25(14):1754–60. 10.1093/bioinformatics/btp324 19451168 PMC2705234

[16] Yang H, Wang K. Genomic variant annotation and prioritization with ANNOVAR and wANNOVAR. Nat Protoc. 2015;10(10):1556–66. 10.1038/nprot.2015.105 26379229 PMC4718734

[17] Untergasser A, Cutcutache I, Koressaar T, Ye J, Faircloth BC, Remm M, et al Primer3—new capabilities and interfaces. Nucleic Acids Res. 2012;40(15):e115 10.1093/nar/gks596 22730293 PMC3424584

[18] den Dunnen JT, Dalgleish R, Maglott DR, Hart RK, Greenblatt MS, McGowan-Jordan J, et al HGVS Recommendations for the Description of Sequence Variants: 2016 Update. Hum Mutat. 2016;37(6):564–9. 10.1002/humu.22981 26931183

[19] Practice Bulletin No. 162: Prenatal Diagnostic Testing for Genetic Disorders. Obstet Gynecol. 2016. 10.1097/AOG.0000000000001405 26938573

[20] Richards S, Aziz N, Bale S, Bick D, Das S, Gastier-Foster J, et al Standards and guidelines for the interpretation of sequence variants: a joint consensus recommendation of the American College of Medical Genetics and Genomics and the Association for Molecular Pathology. Genet Med. 2015;17(5):405–24. 10.1038/gim.2015.30 25741868 PMC4544753

[21] Lek M, Karczewski K, Minikel E, Samocha K, Banks E, Fennell T, et al Analysis of protein-coding genetic variation in 60,706 humans. bioRxiv. 2015. 10.1038/nature19057 PMC5018207 27535533

[22] Genomes Project C, Auton A, Brooks LD, Durbin RM, Garrison EP, Kang HM, et al A global reference for human genetic variation. Nature. 2015;526(7571):68–74. 10.1038/nature15393 26432245 PMC4750478

[23] Maquat LE. Nonsense-mediated mRNA decay: splicing, translation and mRNP dynamics. Nat Rev Mol Cell Biol. 2004;5(2):89–99. 10.1038/nrm1310 15040442

[24] Khajavi M, Inoue K, Lupski JR. Nonsense-mediated mRNA decay modulates clinical outcome of genetic disease. Eur J Hum Genet. 2006;14(10):1074–81. 10.1038/sj.ejhg.5201649 16757948

[25] Scott SA, Edelmann L, Liu L, Luo M, Desnick RJ, Kornreich R. Experience with carrier screening and prenatal diagnosis for 16 Ashkenazi Jewish genetic diseases. Hum Mutat. 2010;31(11):1240–50. 10.1002/humu.21327 20672374 PMC2970726

[26] Jin YY, Zhou W, Tian ZQ, Chen TX. Variable clinical phenotypes of X-linked lymphoproliferative syndrome in China: Report of five cases with three novel mutations and review of the literature. Hum Immunol. 2016;77(8):658–66. 10.1016/j.humimm.2016.06.005 27288720

[27] Sun J, Ying W, Liu D, Hui X, Yu Y, Wang J, et al Clinical and genetic features of 5 Chinese patients with X-linked lymphoproliferative syndrome. Scand J Immunol. 2013;78(5):463–7. 10.1111/sji.12103 23944711

[28] Filipovich AH, Zhang K, Snow AL, Marsh RA. X-linked lymphoproliferative syndromes: brothers or distant cousins? Blood. 2010;116(18):3398–408. 10.1182/blood-2010-03-275909 20660790 PMC2981470

[29] Rigaud S, Fondaneche MC, Lambert N, Pasquier B, Mateo V, Soulas P, et al XIAP deficiency in humans causes an X-linked lymphoproliferative syndrome. Nature. 2006;444(7115):110–4. 10.1038/nature05257 17080092

[30] Sumegi J, Huang D, Lanyi A, Davis JD, Seemayer TA, Maeda A, et al Correlation of mutations of the SH2D1A gene and epstein-barr virus infection with clinical phenotype and outcome in X-linked lymphoproliferative disease. Blood. 2000;96(9):3118–25. 11049992

[31] Pangalos C, Hagnefelt B, Lilakos K, Konialis C. First applications of a targeted exome sequencing approach in fetuses with ultrasound abnormalities reveals an important fraction of cases with associated gene defects. PeerJ. 2016;4:e1955 10.7717/peerj.1955 27168972 PMC4860337

[32] Drury S, Williams H, Trump N, Boustred C, Gosgene, Lench N, et al Exome sequencing for prenatal diagnosis of fetuses with sonographic abnormalities. Prenat Diagn. 2015;35(10):1010–7. 10.1002/pd.4675 26275891

[33] Woon ST, Ameratunga R, Croxson M, Taylor G, Neas K, Edkins E, et al Follicular lymphoma in a X-linked lymphoproliferative syndrome carrier female. Scand J Immunol. 2008;68(2):153–8. 10.1111/j.1365-3083.2008.02128.x 18702745

[34] Rezaei N, Mahmoudi E, Aghamohammadi A, Das R, Nichols KE. X-linked lymphoproliferative syndrome: a genetic condition typified by the triad of infection, immunodeficiency and lymphoma. Br J Haematol. 2011;152(1):13–30. 10.1111/j.1365-2141.2010.08442.x 21083659

